# Remarks on Certain Diseases of the Nails and the Surrounding Soft Parts

**Published:** 1829-02

**Authors:** G. T. Burnett


### THE LONDON
### Medical and Physical Journal.

No. 360, VOL. LXI.] FEBRUARY, 1829. [No. 32, *New Series.*

For many fortunate discoveries in medicine, and for the detection of numerous errors, the world is indebted to the rapid circulation of Monthly Journals; and there never existed any work, to which the Faculty, in Europe and America, were under deeper obligations than to the *Medical and Physical Journal of London*, now forming a long, but an invaluable series.—RUSH.

### ORIGINAL PAPERS AND CASES,

OBTAINED FROM PUBLIC INSTITUTIONS AND OTHER AUTHENTIC SOURCES

### DISEASES OF THE NAILS.

*Remarks on certain Diseases of the Nails and the surrounding soft Parts.* By G. T. BURNETT, Esq.

THERE are certain diseases of the nails, which, from the little notice they have excited, would seem to be rare, and yet which, from the cases I have already met with, I can scarcely believe to be uncommon. It is true that they do not in general endanger life: still they are oftentimes the cause of great and continued suffering. Can it be that professional pride hath led to their proscription, as too trifling to be worthy the surgeon's notice; and, by excluding them from regular, hath consigned them to empirical practice? or can it be that the unsettled treatment and unsatisfactory results of such cases, have been the cause of this apparently contemptuous silence! so that they have scarcely, if at all, a place or name in any of our systems of nosology.

Should the following observations on one of these trifles seem worthy a page in your forthcoming Number, the influence of the London Medical and Physical Journal, which has done much for our very important, though at present imperfect, science, may probably induce some of its readers, as opportunity occurs, to turn their attention to an apparently trifling, yet exceedingly troublesome, form of *onychia* [?] which, I believe, is seldom, if ever, mentioned. Indeed, I scarcely know if I am justified in thus designating the cases to which I refer, as they decidedly possess not either the history or characters of the true or common whitlow. Megonychia, or Onychia ulcerata, might be less exceptionable terms; and, as Paronychia is not only the most familiar, but also the best name for true whitlow, Onychia, often used as a synonym therewith, might not improperly include the two.

The letter of your Paris correspondent, published in the Number for December last, well describes the severe and painful operations resorted to for the cure of onychia maligna; and some of the cases thus called I cannot but think are cases of onychia ulcerata, such as I am going to describe, rather than of the onychia maligna of Mr. Wardrop. If so, the treatment I would recommend is certainly much more mild and simple, though, as far as my limited experience goes, not less efficacious.

The nails, like all other parts of the body, are subject to occasional variations in their form and mode of development, giving rise to preternatural and morbid growths: when these variations are slight, they pass unnoticed; even when greater, if they produce not much inconvenience, they are regarded as harmless monstrosities, rather than disease: when, however, the ordinary duties of organs are disturbed, and especially when pain ensues, the interference of the surgeon is demanded.

The morbid development to which the present observations more particularly refer, is that in which the nail is not only broader than natural, but the sides curve preternaturally downwards, at first compressing, and subsequently entering, the nail bed; causing, in the early stages, pain and inflammation, and afterwards ulcerations, even of a malignant kind. Sometimes the morbid development is of the nail alone, sometimes chiefly of the onythalamus, but most frequently of both: i. e. not only does the nail morbidly incurvate, but the nail bed also is preternaturally enlarged. This disease most commonly affects the feet, especially the great toes: the hands, however, are not exempt, the thumbs occasionally being thus diseased. The detail of a case, however, perhaps may prove the best description.

—— Saunders, a healthy young man, aged twenty, consulted me on account of lameness, occasioned by pain and swelling of the left foot, especially of the great toe. On examination, the toe was found inflamed, being very red, hot, tumid, and painful; the inflammation extended also towards the inner ankle, and laterally over the metatarsus: on any attempt to walk, the placing the foot on the ground produced great uneasiness, and if persevered in, "torture." The right side of the toe was chiefly affected, and from the sulcus between the nail and fillet which bounds the onythalamus there was a semi-purulent discharge; unhealthy granulations had sprung up in the ulcerated cleft; and these, with the ichorous discharge, &c. gave the disease, at first sight, the aspect of onychia maligna.

The previous history was obscure, for it had progressed much; the nail having curved considerably downwards, and the nail bed enlarged, before it produced so great inconvenience as to excite attention. He endured this state of things for some time, suffering more or less, before he sought relief; and then pursued the plan (which I remember being ordered for a similar case, when I was dresser at the hospital,) of introducing pledgets of lint into the cleft between the nail and flesh, thus endeavouring to raise the curvature of the nail, and cause it to overlap the soft parts, instead of the soft parts pressing against its edge. This plan, however, (as I remember also was the case when I dressed the patient before referred to,) caused much uneasiness, without affording any adequate relief: it was, therefore, after a time neglected, and shortly the edges of the nail excoriated the soft parts, and an ulcer was produced; and now the pain on attempting to walk was much increased. The side of the nail was then pared away, which gave some ease; and he had been in the habit, before I saw him, of cutting away the side of the nail with his penknife as far as he could reach; but, as the nail grew, its sharp edge advancing into the tender and enlarged fleshy side of the nail bed, increased the disease, the ulcerated surface became more irritable, and the ichorous discharge before mentioned was the result.

At the first glance, as before stated, this did not look unlike a case of onychia maligna; the sulcus between the nail and soft parts being ulcerated, the ulceration chiefly towards the root of the nail, unhealthy granulations sprouting up at this point, a scanty semipurulent discharge being present, and the surrounding parts in a very irritable state. But the history was totally different; for here the ulceration was the consequence, and not the cause, of the affection of the nail. The other side of the toe gave me a good opportunity, by showing a disposition to the same disease, of watching its progress, and verifying the previous details.

After reducing the inflammation by ordinary means, the ulcerated side was dressed with caustic, &c., and the nail allowed to grow its full width; after which, the attempt was again made to reduce the incurvature of the nail, by placing pledgets of lint between and under its edge and the soft parts; and this was also done from the beginning with the other side. Finding the inconvenience of this plan to be great, and, after a perseverance of several weeks, the benefit, if any, to be very little, some other treatment was to be thought of. Two plans would appear nearly equally feasible: either, 1st, to remove the nail, or at least the incurvating sides, with the corresponding portions of the matrix; or, 2d, to remove the soft parts into which the nail pierced. Had the nail been removed by avulsion, or destroyed by cautery, or dissected out with its matrix, as recommended in onychia maligna, (the operation in this case must have been performed on both sides of the toe, if any part of the nail was to be preserved; and,) to say the least, it would have seemed severe and painful treatment in so apparently trivial a disease. I therefore preferred the latter practice, viz. the removal of the sides of the onythalamus; and, placing the point of a catlin in the cleft, removed at once the side of the nail bed into which the nail entered. The sore soon healed, no cause of irritation being left; and the like operation was subsequently performed on the other side of the toe, with the like result.

I fear I have trespassed as far as the subject will warrant on your valuable pages, and will therefore only add that, when the toe of the other foot of the same person became similarly affected, no time was wasted in palliative treatment, but, by removing the exuberant sides of the onythalamus before ulceration took place, I avoided by anticipation much pain and inconvenience.

Lest, however, I should be misunderstood, let me, in conclusion, state that I am far from thinking this treatment could be beneficial in cases of true onychia maligna, in which (at least as far as I have been able to perceive,) the abscess and ulceration are at and under the root of the nail, the burrowing of which produces the necessity of removing the present nail, but which, the ulceration being cured, would not be injured by the growth of a future one; and, therefore, when I hear of the necessity of dissecting out the matrix, as well as removing the nail, I cannot but think that some cases have been considered as onychia maligna, which in truth were rather onychia ulcerata; as in the former the future growth of the nail would not, and in the latter it would, if the soft parts remained, reproduce the disease.

In a case of onychia maligna, which occurred in a young lady aged twelve, the nail was obliged to be removed, but the matrix was left untouched; the ulcerations were healed, and the nail grew again, but there was no return of the disease, at least not in that finger. This had been a very severe case, and the matrix was so much disturbed that the nail, when reproduced, grew in longitudinal plicæ, or undulations, which, having an unsightly appearance, have since produced much chagrin.

Neither could the removal of the sides of the onythalamus be useful in all cases even of megonychia; for I remember an instance in which the morbid growth and incurvation took place lengthwise, the nail being much thickened as well as curved, assuming an appearance something between an horn and a claw. This monstrosity, which had for years gradually increased, and had twice been cut off, at length was allowed to grow into the flesh halfway down the first phalanx of the thumb; and, when I was consulted, the parts were in such a state of disease that amputation (which accordingly was performed) promised the only chance of relief.

These cases may seem trifling, and scarce worthy of being placed on record; but, shall splendid volumes be published on the great operations of surgery, which can only by few and on few persons be performed, and shall a transitory notice be denied to lesser ills, which, by the frequency of their occurrence, make up the great sum of human misery?

*January 12th*, 1829.

